# Antiviral Susceptibility of Highly Pathogenic Avian Influenza A(H5N1) Viruses Isolated from Poultry, Vietnam, 2009–2011

**DOI:** 10.3201/eid1912.130705

**Published:** 2013-12

**Authors:** Ha T. Nguyen, Tung Nguyen, Vasiliy P. Mishin, Katrina Sleeman, Amanda Balish, Joyce Jones, Adrian Creanga, Henju Marjuki, Timothy M. Uyeki, Dang H. Nguyen, Diep T. Nguyen, Hoa T. Do, Alexander I. Klimov, Charles T. Davis, Larisa V. Gubareva

**Affiliations:** Centers for Disease Control and Prevention, Atlanta, Georgia, USA (H.T. Nguyen, V.P. Mishin, K. Sleeman, A. Balish, J. Jones, H. Marjuki, T.M. Uyeki, A.I. Klimov, C.T. Davis, L.V. Gubareva);; Battelle Memorial Institute, Atlanta (H.T. Nguyen, A. Creanga);; National Center for Veterinary Diagnostics, Hanoi, Vietnam (T. Nguyen, D.H. Nguyen, D.T. Nguyen);; Atlanta Research and Education Foundation, Atlanta (J. Jones)

**Keywords:** Influenza, viruses, H5N1, Oseltamivir, H275Y, antiviral, zoonoses

## Abstract

We assessed drug susceptibilities of 125 avian influenza A(H5N1) viruses isolated from poultry in Vietnam during 2009–2011. Of 25 clade 1.1 viruses, all possessed a marker of resistance to M2 blockers amantadine and rimantadine; 24 were inhibited by neuraminidase inhibitors. One clade 1.1 virus contained the R430W neuraminidase gene and reduced inhibition by oseltamivir, zanamivir, and laninamivir 12-, 73-, and 29-fold, respectively. Three of 30 clade 2.3.4 viruses contained a I223T mutation and showed 7-fold reduced inhibition by oseltamivir. One of 70 clade 2.3.2.1 viruses had the H275Y marker of oseltamivir resistance and exhibited highly reduced inhibition by oseltamivir and peramivir; antiviral agents DAS181 and favipiravir inhibited H275Y mutant virus replication in MDCK-SIAT1 cells. Replicative fitness of the H275Y mutant virus was comparable to that of wildtype virus. These findings highlight the role of drug susceptibility monitoring of H5N1 subtype viruses circulating among birds to inform antiviral stockpiling decisions for pandemic preparedness.

Sporadic transmission of highly pathogenic avian influenza (HPAI) A(H5N1) viruses from birds to humans has been documented since 1997 (*1*), and these viruses continue to cause severe illness and death in humans. Their wide geographic spread and rapid evolution have raised concerns over emergence of a novel, virulent virus that could efficiently transmit among humans, leading to a pandemic. Vietnam is among the countries experiencing the highest number of human fatalities caused by zoonotic H5N1 subtype infections. Since the introduction of HPAI (H5N1) viruses into poultry in Vietnam during 2003 (*1*,*2*), there have been dynamic changes in their genetic and antigenic properties. Clade 1 viruses predominated in Vietnam before 2007, and were the most commonly detected H5N1 subtype group in the Mekong Delta region through 2010 (*3*). However, in northern Vietnam provinces, clade 2.3.4 viruses became the predominant group during 2007–2010. Since 2010, viruses of clade 2.3.2.1 have been detected in poultry from both regions (*3*). Since 2009, multiple subgroups of 2.3.2.1 rapidly emerged and have circulated among domestic poultry in Asia, including several provinces of Vietnam (*4*).

Genetic and antigenic divergence of HPAI (H5N1) viruses among poultry challenges development of effective vaccines for poultry and to pandemic preparedness and development of antiviral drugs for humans. Assessment of drug susceptibility has become an integral part of subtype H5N1 virus surveillance. To assist laboratories worldwide in their surveillance and pandemic preparedness efforts, the Influenza Division of the Centers for Disease Control and Prevention (CDC), Atlanta, GA, USA, along with other partners, developed the H5N1 Genetic Changes Inventory that includes established and potential markers of drug resistance (*5*). Resistance to matrix 2 (M2) protein blockers amantadine and rimantadine, caused by mutations in the M2 protein, is detected commonly in clade 1.1 (S31N) and clade 2.1.3 (V27A) H5N1 virus subtypes and sporadically in other groups (*6*,*7*). Oseltamivir, an orally administered neuraminidase (NA) inhibitor, is the most prescribed medication for the treatment of persons with influenza virus infections. Emergence of resistance to NA inhibitors among H5N1 virus subtypes, especially oseltamivir resistance among H5N1 subtypes caused by the H275Y mutation, is a constant threat (*8*). Assessment of susceptibility to NA inhibitors is hampered by several factors: insufficient knowledge of molecular markers of resistance, lack of harmonized approaches for testing and data analysis and, most critically, lack of established laboratory correlates of clinically relevant resistance. Taking into account these and other limitations, the current method for monitoring susceptibility to NA inhibitors is a critical element needed to evaluate pandemic risk.

In this study, we assessed drug susceptibility profiles of HPAI A(H5N1) viruses isolated from poultry specimens collected in Vietnam during 2009–2011. The antiviral drugs tested included FDA-approved medications and investigational antiviral agents. We report the detection of an oseltamivir-resistant virus with H275Y mutation from the expanding clade 2.3.2.1.

## Materials and Methods

### Viruses

Viruses collected from poultry on farms, in backyard flocks, and in live-poultry markets in Vietnam during 2009–2011 were identified as HPAI A(H5N1) at the National Center for Veterinary Diagnostics (NCVD), Vietnam, by using the World Health Organization (WHO) protocol (*9*). Viruses were then sent to the WHO Collaborating Center for Surveillance, Epidemiology and Control of Influenza at CDC, where they were isolated from eggs and further propagated according to WHO protocol (*9*). Virus handling was conducted under enhanced Biosafety Level 3 containment according to institutional guidelines.

### Sequencing and Phylogenetic Analysis

Full-length gene sequences were generated by the ABI PRISM 3730 Genetic Analyzer (Applied Biosystems, Foster City, CA, USA) and assembled by using Sequencher 5.0 (Gene Codes Corporation, Ann Arbor, MI, USA). Phylogenetic trees were generated by using MEGA version 5.0 (www.megasoftware.net) neighbor-joining methods implemented with 1,000 bootstrap replicates. Phylogenetic data for the strain A/goose/Guangdong/1/1996 (clade 0) were used as a reference for tree rooting and numbering, and trees were annotated according to the WHO/World Organisation for Animal Health/Food and Agriculture Organization of the United Nations criteria (*10*). Sequences were deposited into the Global Initiative on Sharing All Influenza Data database. Accession numbers are listed in Technical Appendix 1. For NA sequences, N1 aa numbering is used throughout the text (*11*). The pyrosequencing method was used to detect NA residue 275 in inoculated ferret nasal wash samples (*12*).

**Table 1:** Clade-specific analysis of drug susceptibility of highly pathogenic avian Influenza A(H5N1) viruses in the neuraminidase inhibition assay*

Neuraminidase inhibitor	Clade	No.	IC_50_ (nmol/L)				Baseline‡			Mild outliers§			Extreme outliers¶	
			Min-Max	Median	Mean±SD†		No.	Min-Max		No.	IC_50_		No.	IC_50_
Oseltamivir	1.1	25	0.04–0.69	0.06	0.07 ± 0.02		23	0.04–0.16		1	0.52		1	0.69
	2.3.2.1	70	0.13–527.26	0.41	0.43 ± 0.17		67	0.13–0.87		2	1.72–2.01		1	527.26
	2.3.4	28	0.51–11.36	1.62	1.48 ± 0.74		24	0.21–2.79		4	6.76–11.36		0	NA
	2.3.4/R**	2	0.48–0.52	NA ^‡‡^	0.50 ± 0.10		2	0.48–0.52		0	NA		0	NA
Zanamivir	1.1	25	0.13–18.89	0.26	0.28 ± 0.10		23	0.13–0.54		1	1.33		1	18.89
	2.3.2.1	70	0.14–1.11	0.32	0.34 ± 0.12		68	0.14–0.68		1	1.11		0	NA
	2.3.4	28	0.21–1.71	0.51	0.52 ± 0.18		27	0.21–0.99		1	1.71		0	NA
	2.3.4/R	2	0.48–0.62	NA	0.55 ± 0.07		2	0.48–0.62		0	NA		0	NA
Peramivir	1.1	25	0.07–0.31	0.13	0.14 ± 0.03		25	0.07–0.31		0	NA		0	NA
	2.3.2.1	70	0.09–91.22	0.20	0.21 ± 0.09		69	0.09–0.47		0	NA		1	91.22
	2.3.4	28	0.16–0.52	0.29	0.31 ± 0.10		30	0.16–0.52		0	NA		0	NA
	2.3.4/R	2	0.28–0.39	NA	0.33 ± 0.05		2	0.28–0.39		0	NA		0	NA

*IC _50_, 50% inhibitory concentration; No., number of viruses analyzed; Min-Max, minimum to maximum of IC_50_ values; NA, not applicable; IQR, interquartile range.  †Mean and SD of IC_50_ values after exclusion of outliers. ‡ Viruses with IC_50_ values <U = Q3+3.0*(IQRIQR= Q3-Q1; Q1 = 25th percentile; Q3= 75th percentile. §Mild outliers, with IC_50_ >U but <10-fold difference compared with the median IC_50_. ¶Extreme outliers, with IC_50_ >U and ≥10-fold difference compared with the median IC_50_. **2.3.4/R,reassortants; the viruses belong to clade 2.3.4 but the neuraminidase genes are grouped with clade 2.3.2.1 (also see Technical Appendix, Table 2).

### NA Inhibitors, Neuraminidase Inhibition Assay, and 50% Inhibitory Concentration Analysis

Susceptibility to the drugs zanamivir (GlaxoSmithKline, Uxbridge, UK), oseltamivir (Roche Diagnostics GmbH, Mannheim, Germany), peramivir (BioCryst Pharmaceuticals, Birmingham, AL, USA), and laninamivir (compound R-125489; Biota, Begbroke, UK) was assessed by fluorescent neuraminidase inhibition (NI) assay, by using inhibitor concentrations ranging from 0.03 nmol/L to 1,000 nmol/L (*13*). The 50% inhibitory concentration (IC_50_) values, the drug concentration needed to inhibit virus NA activity by 50%, were determined by using a CDC in-house program, the JASPR v1.2 curve-fitting software (*14*). Statistical analysis of IC_50_ values was performed by using SAS 9.2 software (SAS Institute, Cary, NC, USA) to identify outliers, using a statistical cutoff value U = Q3+3.0*(interquartile range). The interquartile range was determined as Q3–Q1; Q1 or Q3 denoted 25th or 75th percentile, respectively. The resulting value was applied for clade and drug. Mild outliers were defined as viruses that had IC_50_ values >U and <10 times the median, and extreme outliers as having IC_50_values >U and ≥10 times the median. SigmaPlot 12 (Systat Software, Chicago, IL, USA) was used to generate box-and-whisker plots to visualize outliers. The median/mean IC_50_ values among virus clades were analyzed by using the Kruskal-Wallis 1-way analysis of variance and the Dunn’s multiple comparison test, respectively.

### Susceptibility to Antiviral Agents in Cell Culture

Susceptibilities to the M2 blocker amantadine and to investigational agents DAS181 and favipiravir (T705) were assessed in a virus yield reduction assay on Madin-Darby canine kidney (MDCK) SIAT1 cells (*15*,*16*). In brief, the confluent cell monolayers seeded on 96-well plates were treated before inoculation with amantadine or favipiravir for 30 minutes or with DAS181 for 2 hours. After the drug was removed, cells were inoculated with either wildtype (WT) virus, which did not contain the H275Y mutation in NA, or oseltamivir-resistant virus with the H275Y mutation (as positive control) at a low multiplicity of infection of 0.0001 PFU/cell and incubated for 1 hour at 4°C. Cells were washed and added to fresh media containing drug dilutions, and incubated at 37°C for 24 hours. Supernatants were harvested to determine infectious virus yield 50% tissue culture infectious dose per mL (TCID_50_/mL) in MDCK-SIAT1 cells. The 90% effective concentration (EC_90_) of drug (drug concentration that reduces the infectious virus yield by 90%) was determined by using the 4-parameter logistic nonlinear regression model equation in GraphPad Prism 5 software (GraphPad Software, La Jolla, CA, USA).

### Replicative Capacity in Cell Culture and Ferret Upper Respiratory Tract

Comparison of replicative capacity of WT and oseltamivir-resistant H275Y mutant virus was achieved by inoculating MDCK-SIAT1 cells at a multiplicity of infection of 0.0001 PFU/cell in 24-well plates. Cells were incubated at 37°C, and supernatants were collected every 12 hours until 72 hours postinoculation. At each time point, the infectious virus yields (TCID_50_/mL) were determined by titrating the supernatants on MDCK-SIAT1 cells (*17*). In vivo replicative capacity was assessed in ferrets by inoculating naïve, anesthetized animals, 3–5 months of age, with 10^6^ TCID_50_ of either WT or H275Y virus. Virus titers (TCID_50_/mL) were measured in the nasal washes collected postinoculation on days 1–3, 5, 7, and 9.

## Results

### Phylogenetic Analysis and Resistance Markers

**Figure 1:**
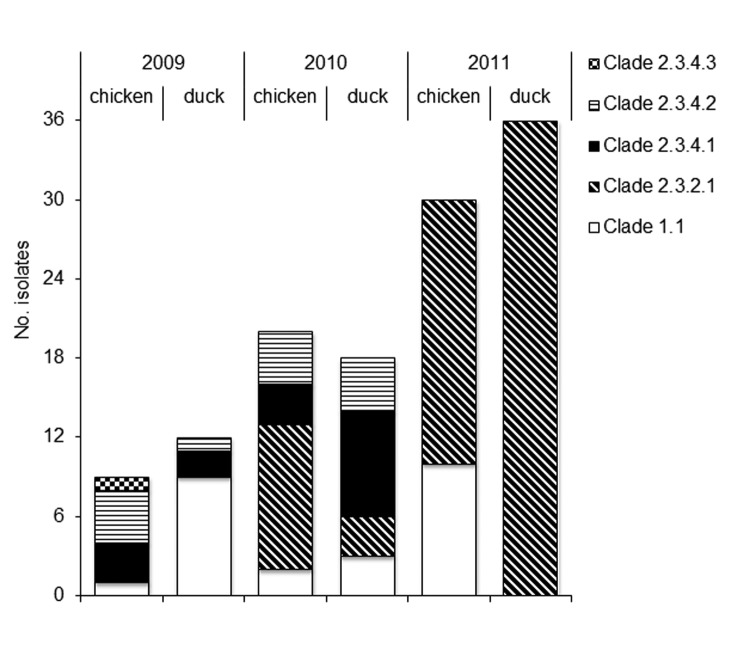
Description of 125 highly pathogenic avian influenza A(H5N1) viruses collected from poultry in Vietnam during 2009–2011 and tested during this study.

**Figure 2:**
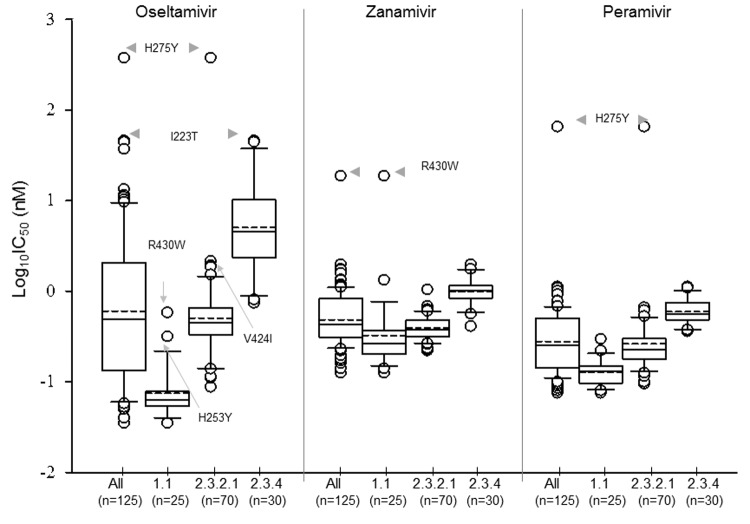
Distribution of log-transformed 50% inhibitory concentration (IC_50_) values for oseltamivir, zanamivir, and peramivir: Box-and-whisker plot analysis of all tested highly pathogenic avian influenza A(H5N1) viruses (n = 125) and individual clade for each virus. The boxes represent the 25th (quartile 1) to 75th (quartile 3) percentiles; horizontal and dash lines within the box represent median and mean values, respectively; n, number of viruses tested.

Knowledge of subtype H5N1 virus genomics, especially that of hemagglutinin (HA), NA, and membrane protein, or M genes, is required for interpretation of drug susceptibility data and for uncovering new trends. According to the HA gene phylogeny, viruses isolated from poultry in Vietnam during 2009–2011 were assigned to 3 clades: 1.1 (n = 25), 2.3.2.1 (n = 70), and 2.3.4 (n = 30) (Figure 1; Technical Appendix 2, Figure 2, panel A. The viruses of clade 2.3.4 were categorized as 3 subclades, termed 2.3.4.1, 2.3.4.2, and 2.3.4.3. The NA phylogenetic tree topology (Technical Appendix 2, Figure 2, panel B) was comparable to the HA tree for clades 1.1 and 2.3.2.1, but not for clade 2.3.4 viruses, suggesting NA gene reassortment among these viruses. Two reassortant viruses of subclade 2.3.4.1 contained NA genes similar to those of subclade 2.3.2.1 viruses. 

The M tree was similar to the HA tree and showed evidence of reassortment for a single clade 2.3.4.2 virus that clustered with M genes from subclade 2.3.4.1 (Technical Appendix 2, Figure 2, panel C). The H5N1 Genetic Changes Inventory (*5*) was used to screen NA and M gene alignments for molecular markers associated with potential drug resistance. Clade 1.1 viruses contained S31N in M2 protein, the most common marker of M2 blocker resistance. This mutation was present in combination with L26I, a typical feature of clade 1 and 1.1 viruses. The remaining viruses had no known markers of M2 blocker resistance. In the NA gene, the H275Y mutation, a marker of oseltamivir-resistance, was detected in the virus A/duck/VN/NCVD-664/2010 (clade 2.3.2.1). The virus was isolated in Ninh Binh Province in northern Vietnam. In subclade 2.3.4.2 (n = 13), 3 viruses contained the mutation I223T, which may affect susceptibility to NA inhibitors. One clade 1.1 virus carried the mutation V149A, which was previously linked to slightly reduced susceptibility to zanamivir (*18*).

### Drug Susceptibility in NI Assay

To identify viruses with potential resistance to NA inhibitor(s), we performed the NI assay using oseltamivir, zanamivir, and peramivir. IC_50_ values were calculated for each virus and drug. The median IC_50_ values were similar for oseltamivir and zanamivir (0.44 nmol/L and 0.36 nmol/L, respectively) and ≈2-fold lower for peramivir (Technical Appendix 2, Table). The influence of the NA sequence diversity on IC_50_ values was most noticeable from the wide-ranging oseltamivir IC_50_ values (>13,000-fold difference between minimum and maximum), whereas the range was much narrower for zanamivir (145-fold) and intermediate for peramivir (1,300-fold). Analysis of IC_50_ values was further achieved by individual clade to enable better correlation with NA sequences (Table 1; Figure 2). The 2 reassortants were excluded from clade 2.3.4 analysis because the associated NA gene from each was related to those from clade 2.3.2.1.

The median oseltamivir IC_50_ value for clade 1.1 was lower than that of clades 2.3.2.1 and 2.3.4, by 7- to 27-fold, respectively. In clade 1.1, virus A/chicken/Vietnam/NCVD-780/2011 was identified as an extreme outlier and showed a 12-fold increased oseltamivir IC_50_ value (Table 2; Figure 2). This virus contained a previously unreported change: the presence of NA mutation R430W. Virus A/chicken/Vietnam/NCVD-776/2011 was identified as a mild outlier: it had the H253Y mutation and showed a 9-fold increase in oseltamivir IC_50_ values; virus A/chicken/Vietnam/NCVD-878/2011, which carried V149A, exhibited a 3-fold increase. Notably, the oseltamivir IC_50_ values of all 3 outliers described were similar to, or less than, the median oseltamivir IC_50_ value of clade 2 viruses (Tables 1, 2; Figure 2).

**Table 2:** Characterization of statistical outliers identified in neuraminidase inhibition assay*

Clade	Virus name	Neuraminidase gene change†	IC_50_**_,_** nmol/L; mean±SD (fold)‡			
			Oseltamivir	Zanamivir	Peramivir	Laninamivir
1.1	A/ck/VN/NCVD-780/2011	R430W	0.69 ± 0.25 (12)	18.89 ± 2.18 (73)	0.29 ± 0.03 (2)	2.62 ± 0.02 (29)
	A/ck/VN/NCVD-878/2011	V149A	0.16 (3)	1.33 (5)	0.13 (1)	NT
	A/ck/VN/NCVD-776/2011	H253Y	0.52 ± 0.24 (9)	0.20 ± 0.03 (1)	0.10 ± 0.03 (1)	0.14 ± 0.01 (1)
	Median	NA	0.06	0.26	0.13	0.09 ± 0.01§
2.3.2.1	A/dk/VN/NCVD-664/2010	H275Y	527.26 ± 201.10 (1,353)	1.11 ± 0.67 (3)	91.22 ± 44.34 (415)	1.36 ± 0.72 (6)
	A/dk/VN/NCVD-712/2011	V424I	2.01 (5)	0.52 (2)	0.26 (1)	NT
	A/dk/VN/NCVD-714/2011	V424I	1.72 (4)	0.53 (2)	0.31(1)	NT
	Median	NA	0.41	0.32	0.2	0.23 ± 0.01§
2.3.4	A/ck/VN/NCVD-296/2009	I223T	10.99 ± 2.38 (7)	0.86 ± 0.33 (2)	0.52 ± 0.17 (2)	0.49 ± 0.24
	A/ck/VN/NCVD-295/2009	I223T	10.37 ± 1.78 (6)	0.93 ± 0.19 (2)	0.50 ± 0.13 (2)	0.53 ± 0.18
	A/ck/VN/NCVD-283/2009	I223T	11.36 ± 3.43 (7)	0.77 ± 0.28 (2)	0.49 ± 0.24 (2)	0.42 ± 0.16
	A/dk/VN/NCVD-462/2010	G147R	6.76 ± 0.44 (4)	1.71 ± 0.48 (3)	0.45 ± 0.06 (2)	0.40 ± 0.17
	Median	NA	1.62	0.51	0.31	0.12 ± 0.02^§^
1	*H5N1* Reference viruses					
	A/VN/HN30408/2005, clone	H275Y	155.18 ± 5.77 (1,552)	0.63 ± 0.12 (1)	10.88 (64)	1.13 (6)
	A/VN/HN30408/2005, clone	N295S	2.99 ± 0.21 (30)	0.73 (2)	0.13 (1)	0.52 (3)
	A/Vietnam/1203/2004	NA	0.10 ± 0.02 (1)	0.46 ± 0.06	0.17 ± 0.03	0.18 ± 0.03
NA	*H1N1pdm09* Reference viruses					
	A/North Carolina/39/2009	H275Y	138.06 ± 26.02 (727)	0.19 ± 0.03 (1)	16.77 ± 4.47 (335)	0.26 ± 0.05 (1)
	A/California/07/2009		0.19 ± 0.05 (1)	0.18 ± 0.02	0.05 ± 0.01	0.17 ± 0.04

* IC _50_, 50% inhibitory concentration; NT, not tested; NA, not applicable. †Compared with the neuraminidase gene sequence of the closest match. (See Table 1 for median IC_50_ for each clade.) ‡Fold increase compared with the median IC_50_ of the same clade virus. §Fold increase compared with the IC_50_ of the closest matching virus in the same clade. Global Initiative on Sharing All Influenza Data NA accession no. shown in Technical Appendix 1.

Influenza virus strain H5N1 A/duck/Vietnam/NCVD-664/2010 was identified as an extreme outlier for oseltamivir susceptibility in clade 2.3.2.1; it contained the marker H275Y and exhibited a 1,353-fold elevation in IC_50_. Two mild outliers (3–5-fold increase) that carried the V424I change were identified within the same clade. In clade 2.3.4 viruses, 4 outliers for oseltamivir were detected, 3 of which possessed I223T, which conferred a 6–7-fold increase in IC_50_ values. The fourth virus had a V147R substitution and exhibited a 4-fold increase in IC_50_ (Table 2). As anticipated from the results of phylogenetic analysis, oseltamivir IC_50_ values of the 2 reassortant viruses (HA of clade 2.3.4 but NA from clade 2.3.2.1) matched those of clade 2.3.2.1 viruses (Table 1).

When tested for zanamivir susceptibility, an extreme outlier that had a 73-fold increase in IC_50_ was detected in clade 1.1 (Table 2): this was the same virus, A/chicken/Vietnam/NCVD-780/2011, that showed a previously unknown R430W change and was identified as an extreme outlier for oseltamivir susceptibility. Three mild outliers were identified from clades 1.1, 2.3.2.1, and 2.3.4 and had amino acid changes at the V149A, H275Y, and G147R substitutions, respectively.

The virus A/duck/Vietnam/NCVD-664/2010 that carried the H275Y mutation was predictably identified as an extreme outlier for peramivir with a 415-fold increase in IC_50_ values; the remaining viruses showed no increase. Among a subset of viruses (n = 38) tested with laninamivir, the virus that carried the R430W mutation showed a 29-fold increase, and the virus that had the H275Y mutation showed a 6-fold increase in IC_50_ values.

The WHO criteria for reporting NI assay data for influenza viruses (*19*) are based on fold difference between IC_50_ values of the test virus and a reference IC_50_ value (such as median IC_50_); different criteria are set for seasonal type A and type B viruses. The reporting for H5N1subtypes is not specified; therefore, we followed the criteria as outlined for seasonal type A viruses, but grouped the IC_50_ values by clade (Table 1). For clade 1.1, the virus that had the R430W mutation showed reduced inhibition by oseltamivir, zanamivir, and laninamivir; in clade 2.3.2.1, the virus that had the H275Y mutation showed highly reduced inhibition by oseltamivir and peramivir.

### Characterization of the Oseltamivir-Resistant H275Y Virus

**Figure 3:**
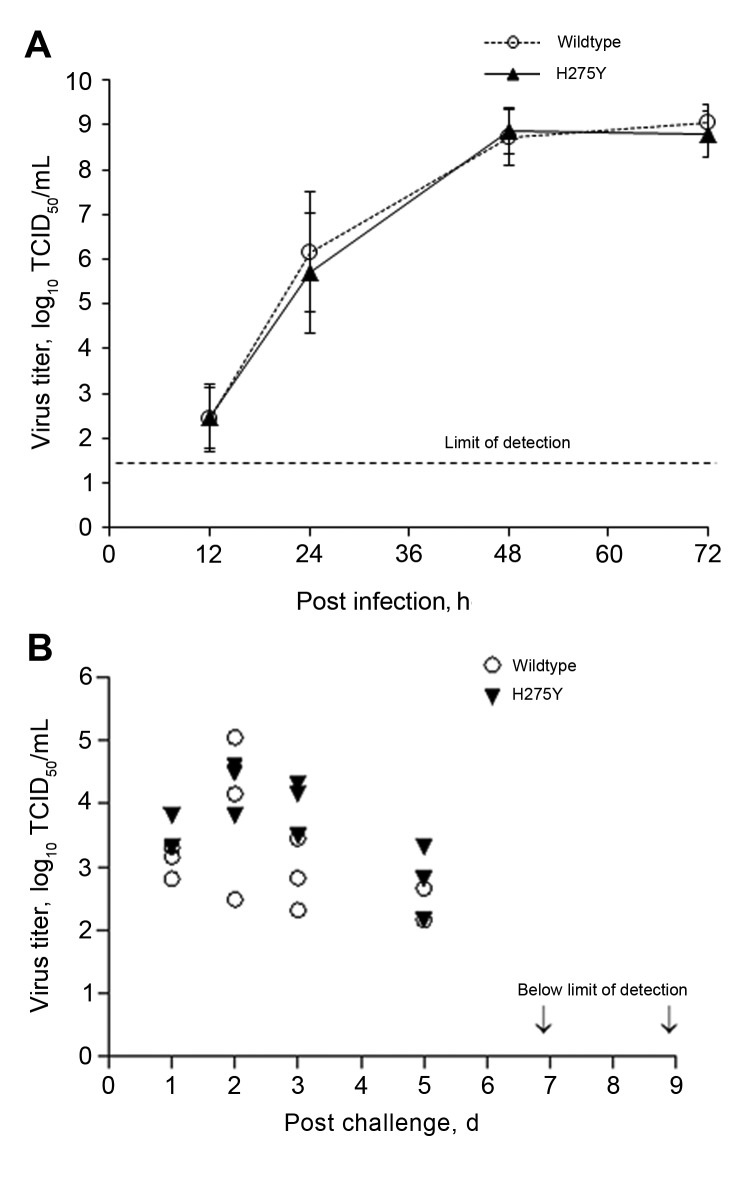
Replicative capacity of the oseltamivir-resistant highly pathogenic avian influenza A(H5N1) virus possessing the H275Y substitution and the wild type virus in (A) MDCK and MDCK-SIAT1 cell lines and (B) in the ferret upper respiratory tract; nasal washes were collected on days 1, 2, 3, 5, 7, and 9 post challenge. Of note, the limit of detection for virus titer was set at 1.3 x log_10._

The oseltamivir-resistant virus was also tested with antiviral agents with mechanisms of action other than NA inhibition. The infectious virus yields of WT and the oseltamivir-resistant virus were reduced by >2 logs at 1 µg/mL of amantadine (data not shown), which is consistent with the M2 blocker–sensitive genotype. Inoculation of cells with DAS181 before incubation was equally effective in inhibiting replication of the virus with H275Y mutation and the WT virus (Table 3). Both viruses were equally susceptible to favipiravir, expressing EC_90_ values of 3 µmol/L–6 µmol/L (Table 4). For risk assessment, it was essential to investigate whether the H275Y mutation had a detrimental effect on virus replication. In MDCK-SIAT1 cells, the H275Y-mutated virus replicated at a similar rate as the WT virus and reached infectious titers as high as 10^9^ TCID_50_/mL at 72 hours post infection (Figure 3, panel A). In the ferret model, the H275Y virus titers in the nasal washes collected at several points after inoculation were similar to those of the WT virus (Figure 3, panel B) and no differences in symptoms were noted. The stability of the H275Y mutation was demonstrated by analyzing pyrosequencing data of the viruses shed by the infected ferrets.

**Table 3:** Reduction of virus yield in MDCK-SIAT1 cells in the presence of antiviral agent DAS181*

H5N1 subtype	NA	Mean±SD virus yield (log_10_TCID_50_/mL), DAS181 (µmol/L)						EC_90_ (µmol/L), mean±SD
0	0.04	0.16	0.63	2.5	10
A/dk/VN/NCVD-680/2011	Wildtype	6.6 ± 0.7	5.3 ± 0.7	3.3 ± 0.5	3±0	†	†	0.02 ± 0.01
A/dk/VN/NCVD-664/2010	H275Y	7.0 ± 0.9	4.6 ± 0.8	4.3 ± 0.9	†	†	‡	0.01 ± 0.01

*Multiplicity of infection 0.0001/cell in 24-well plate; NA, neuraminidase; TCID _50_/mL, 50% tissue culture infectious dose; EC_90_, 90% effective concentration.  †Below the limit of detection, 1.4 log_10_/mL.

**Table 4:** Reduction of virus yield in MDCK-SIAT1 cells in the presence of favipiravir*

H5N1 subtype	NA	Mean±SD virus yield (log_10_TCID_50_/mL;), favipiravir (µmol/L)						EC_90_ (µmol/L), mean±SD
		0	0.4	1.6	6.3	25	100	
A/dk/VN/NCVD-680/2011	wildtype	6.9 ± 0.6	6.9 ± 0.5	5.7 ± 0.7	6.3 ± 0.4	3.8 ± 0.9	†	3.2 ± 2.4
A/dk/VN/NCVD-664/2010	H275Y	7.6 ± 0.5	6.9 ± 0.8	7.5 ± 0.5	6.9 ± 0.7	5.4 ± 0.8	†	5.9 ± 3.2

*Multiplicity of infection 0.0001/cell in 24-well plate; TCID_50_/mL, 50% tissue culture infectious dose; EC_90_, 90% effective concentration; NA, neuraminidase.  †Below the limit of detection, 1.4 log_10_/mL.

## Discussion

In this study, we identified a single oseltamivir-resistant virus among 125 HPAI A(H5N1) viruses isolated from poultry in Vietnam during 2009–2011. It was recovered from a domestic duck in Ninh Binh Province, northern Vietnam. This virus belonged to the rapidly expanding clade 2.3.2.1 and contained the H275Y mutation, which is the principal marker of oseltamivir resistance in N1-subtype viruses (*11*). We observed no impairment in its replicative fitness in either cell culture or a ferret model. Our findings are in agreement with previous studies in which the reverse genetically engineered H275Y mutant from clade 1 retained its in vitro replicative efficiency and high pathogenicity in animals (*20*). Emergence of clade 1 viruses carrying the H275Y mutation was reported in oseltamivir-treated patients (*21*,*22*). When tested in ferrets, replication of the H275Y mutant virus was not inhibited by oseltamivir (*23*), confirming its oseltamivir-resistant phenotype. Of the 3,215 subtype H5N1 virus NA sequences available in GenBank, 6 contain H275Y; these viruses include a virus isolated from a patient treated with oseltamivir in Vietnam during 2005 (*21*), a patient in Indonesia (GenBank accession no. EU146786; unknown treatment history), and 4 viruses isolated from birds in Hong Kong (GenBank accession no. DQ250158) and Russia (GenBank accession no. DQ840522, DQ320136, and CY063862) (*7*). Although detection of H275Y mutations in H5N1 subtypes is rare, the global spread of subtype H1N1 viruses carrying the same mutation in the absence of drug exposure serves as a sobering reminder of the unpredictable nature of influenza virus evolution. As indicated in this and other studies, the HPAI (H5N1) NA gene is subject to reassortment between different HA clade-bearing viruses, which could accelerate this process (*3*).

Apart from the H275Y marker, it is difficult to predict the effect of natural genetic variation in HPAI (H5N1) viruses on susceptibility to NA inhibitors in humans. The range of H5N1 subtype oseltamivir IC_50_ values was wide (0.04–527.26 nmol/L), similar to previous findings (*24*). The WHO criteria for reporting NI assay data are based on a fold increase in the IC_50_ value of a test virus compared with that of a control or reference virus (or median) IC_50_ value (*18*). For influenza A, the result is interpreted as normal (<10-fold), reduced (10- to100-fold), or highly reduced inhibition (>100-fold). 

In this report, the subtype H5N1 influenza virus with the H275Y NA mutation exhibited highly reduced inhibition by oseltamivir and peramivir, and normal inhibition by zanamivir and laninamivir. The current criteria need further clarification when applied to highly diverse H5N1 subtypes. For instance, oseltamivir IC_50_ values of clade 1.1 viruses were 7–27-fold lower than those of clade 2 viruses; this finding is in accord with previous reports (*25*). This difference stems from the presence of either histidine (clade 2) or tyrosine (clade 1) at position 253 in the NA protein (*26*,*27*). Predictably, the identified clade 1.1 virus carrying the revertant H253Y mutation exhibited ≈9-fold higher IC_50_ values compared with the median IC_50_ value for this clade and ≈10-fold increase compared with A/ck/VN/NCVD-777/2011, a matching virus in which the only difference in the NA gene was an H253Y substitution. Such a virus would be reported as exhibiting reduced inhibition, yet its oseltamivir IC_50_ value was below the median IC_50_ value of typical clade 2.3.4 viruses tested in this study. In view of these findings, it may be more appropriate to consider all clade 1 viruses possessing histidine at position 253 as hypersensitive and use a median IC_50_ value of clade 2 viruses in fold difference calculations. If this rule were applied, 1 of the reference H5N1 subtype viruses (clade 1), possessing N295S (*23*), would increase by ≈2-fold in oseltamivir IC_50_ values (2.99 nmol/L) compared with values of clade 2.3.4 viruses (1.62 nmol/L) and, thus, would be reported as exhibiting normal inhibition. Like the H275Y-mutated viruses previously identified, this variant has also been isolated from an oseltamivir-treated patient (*22*) and its replication was not affected by oseltamivir treatment in ferrets (*23*). Therefore, it is essential to be able to detect and report these NA variants (*28*), despite an overall low IC_50_ value.

Because of lack of established laboratory correlates of clinically relevant resistance, analysis and interpretation of IC_50_ values generated in this study were completed according to the WHO criteria with a stipulation that fold change comparison was performed by using median IC_50_ values for individual clades. This approach facillitated identification of variants, such as the R430W-mutated virus from clade 1.1, which should be further studied. Certain discrepancies among reports are to be expected in the absence of standardized assays and criteria. For example, variant V149A of clade 1.1 was reported here as normally inhibited by zanamivir; such a variant was previously reported as showing mildly decreased susceptibility to zanamivir (*18*,*29*). Similarly, substitutions at variant I223 have previously been associated with reduced susceptibility to oseltamivir, zanamivir, or both (*5*). In this study, 3 clade 2.3.4 viruses carrying variant I223T were reported to show normal inhibition by NA inhibitors because of the relatively high median IC_50_ values of this clade. Nevertheless, the oseltamivir IC_50_ values in this clade were ≈55-fold greater than the reference WT A(H1N1)pdm09 virus. Acquired additional changes in the NA (e.g., H275Y) may confer a higher level of resistance to the NA-inhibitor class of drugs among these viruses (*30*,*31*).

HPAI (H5N1) viruses resistant to M2 blockers are prevalent among poultry throughout Asia, including Vietnam (*7*,*32*). Most of these M2-resistant viruses belong to clade 1.1 (*32*,*33*), but they have also been found in other clades, including clade 2.3.4 (*34*). The oseltamivir-resistant H275Y-mutant virus detected in this study was sensitive to M2 blockers; however, the ease with which viruses can acquire resistance to this class of drugs emphasizes the need for alternative therapeutic options. The NA activity of the oseltamivir-resistant H275Y-mutant virus was inhibited by zanamivir and laninamivir in the NI assay. These NA inhibitors are delivered by inhalation, which limits their use for treatment of severely ill patients; an intravenous formulation of zanamivir is in clinical trial and is available on a compassionate use basis for treatment of hospitalized influenza patients (*35*,*36*). The replication of the H275Y-mutant virus and its WT counterpart were equally inhibited by investigational drugs DAS181 and favipiravir in cell culture.

## Conclusion

Our findings demonstrate the critical role of ongoing monitoring of antiviral drug susceptibility in HPAI (H5N1) viruses sampled from poultry on informing antiviral stockpiling decisions for pandemic preparedness. Because 15 countries have reported human cases of HPAI (H5N1) virus infection to date, these findings also emphasize the need to enhance the armamentarium of available anti-influenza drugs worldwide for treatment of subtype H5N1-infected patients, including agents with diverse mechanisms of action, which could enable combination treatment (*37*), and host-directed antiviral therapy, and which may be less vulnerable to resistance.

Technical Appendix 1 Drug susceptibility and accession data for highly pathogenic avian influenza A(H5N1) viruses.

Technical Appendix 2 Analysis of highly pathogenic avian influenza A(H5N1) viruses.

## References

[1] Wan XF, Nguyen T, Davis CT, Smith CB, Zhao ZM, Carrel M, Evolution of highly pathogenic H5N1 avian influenza viruses in Vietnam between 2001 and 2007. PLoS ONE. 2008;3:e3462. 10.1371/journal.pone.0003462 18941631 PMC2565130

[2] Dung Nguyen T, Nguyen TV, Vijaykrishna D, Webster RG, Guan Y, Malik Peiris JS, Multiple sublineages of influenza A virus (H5N1), Vietnam, 2005–2007. Emerg Infect Dis. 2008;14:632–6. 10.3201/eid1404.071343 18394281 PMC2570938

[3] Nguyen T, Rivailler P, Davis CT. Hoa do T, Balish A, Dang NH, et al. Evolution of highly pathogenic avian influenza (H5N1) virus populations in Vietnam between 2007 and 2010. Virology. 2012;432:405–16. 10.1016/j.virol.2012.06.021 22818871

[4] World Health Organization. Antigenic and genetic characteristics of zoonotic influenza viruses and development of candidate vaccine viruses for pandemic preparedness. Influenza, Vaccines, Vaccine Viruses. February 2013 [cited 2013Aug 6]. http://www.who.int/influenza/vaccines/virus/characteristics_virus_vaccines/en/

[5] Centers for Disease Control and Prevention. H5N1 Genetic changes inventory. [cited 2013 Aug 6]. http://www.cdc.gov/flu/avianflu/h5n1/inventory.htm

[6] Cheung CL, Rayner JM, Smith GJ, Wang P, Naipospos TS, Zhang J, Distribution of amantadine-resistant H5N1 avian influenza variants in Asia. J Infect Dis. 2006;193:1626–9. 10.1086/504723 16703504

[7] Hill AW, Guralnick RP, Wilson MJ, Habib F, Janies D. Evolution of drug resistance in multiple distinct lineages of H5N1 avian influenza. Infect Genet Evol. 2009;9:169–78. 10.1016/j.meegid.2008.10.006 19022400

[8] Moscona A. Oseltamivir resistance—disabling our influenza defenses. N Engl J Med. 2005;353:2633–6. 10.1056/NEJMp058291 16371626

[9] World Health Organization Global Influenza Surveillance Network. Manual for the laboratory diagnosis and virological surveillance of influenza. 2011 [cited 2013 Aug 6]. http://whqlibdoc.who.int/publications/2011/9789241548090_eng.pdf

[10] World Health Organization/ World Organisation for Animal Health/ Food and Agriculture Organization (WHO/OIE/FAO) H5N1 Evolution Working Group. Continued evolution of highly pathogenic avian influenza A (H5N1): updated nomenclature. Influenza Other Respi Viruses. 2012;6:1–5 . 10.1111/j.1750-2659.2011.00298.x PMC5074649 22035148

[11] Nguyen HT, Fry AM, Gubareva LV. Neuraminidase inhibitor resistance in influenza viruses and laboratory testing methods. Antivir Ther. 2012;17:159–73. 10.3851/IMP2067 22311680

[12] Deyde VM, Nguyen T, Bright RA, Balish A, Shu B, Lindstrom S, Detection of molecular markers of antiviral resistance in influenza A (H5N1) viruses using a pyrosequencing method. Antimicrob Agents Chemother. 2009;53:1039–47. 10.1128/AAC.01446-08 19124660 PMC2650582

[13] Nguyen HT, Sheu TG, Mishin VP, Klimov AI, Gubareva LV. Assessment of pandemic and seasonal influenza A (H1N1) virus susceptibility to neuraminidase inhibitors in three enzyme activity inhibition assays. Antimicrob Agents Chemother. 2010;54:3671–7. 10.1128/AAC.00581-10 20585136 PMC2934949

[14] Okomo-Adhiambo M, Sleeman K, Ballenger K, Nguyen HT, Mishin VP, Sheu TG, Neuraminidase inhibitor susceptibility testing in human influenza viruses: a laboratory surveillance perspective. Viruses. 2010;2:2269–89. 10.3390/v2102269 21994620 PMC3185571

[15] Gubareva LV, Trujillo AA, Okomo-Adhiambo M, Mishin VP, Deyde VM, Sleeman K, Comprehensive assessment of 2009 pandemic influenza A (H1N1) virus drug susceptibility in vitro. Antivir Ther. 2010;15:1151–9. 10.3851/IMP1678 21149922

[16] Triana-Baltzer GB, Gubareva LV, Klimov AI, Wurtman DF, Moss RB, Hedlund M, Inhibition of neuraminidase inhibitor-resistant influenza virus by DAS181, a novel sialidase fusion protein. PLoS ONE. 2009;4:e7838. 10.1371/journal.pone.0007838 19893749 PMC2770896

[17] Klimov A, Balish A, Veguilla V, Sun H, Schiffer J, Lu X, Influenza virus titration, antigenic characterization, and serological methods for antibody detection. Methods Mol Biol. 2012;865:25–51. 10.1007/978-1-61779-621-0_3 22528152

[18] Naughtin M, Dyason JC, Mardy S, Sorn S, von Itzstein M, Buchy P. Neuraminidase inhibitor sensitivity and receptor-binding specificity of Cambodian clade 1 highly pathogenic H5N1 influenza virus. Antimicrob Agents Chemother. 2011;55:2004–10. 10.1128/AAC.01773-10 21343450 PMC3088255

[19] World Health Organization. Meetings of the WHO working group on surveillance of influenza antiviral susceptibility– Geneva, November 2011 and June 2012. Wkly Epidemiol Rec. 2012;87:369–74 . 23061103

[20] Yen HL, Ilyushina NA, Salomon R, Hoffmann E, Webster RG, Govorkova EA. Neuraminidase inhibitor-resistant recombinant A/Vietnam/1203/04 (H5N1) influenza viruses retain their replication efficiency and pathogenicity *in vitro* and *in vivo.* J Virol. 2007;81:12418–26. 10.1128/JVI.01067-07 17855542 PMC2169015

[21] de Jong MD, Tran TT, Truong HK, Vo MH, Smith GJ, Nguyen VC, Oseltamivir resistance during treatment of influenza A (H5N1) infection. N Engl J Med. 2005;353:2667–72 . 10.1056/NEJMoa054512 16371632

[22] Le QM, Kiso M, Someya K, Sakai YT, Nguyen TH, Nguyen KH, Avian flu: isolation of drug-resistant H5N1 virus. Nature. 2005;437:1108. 10.1038/4371108a 16228009

[23] McKimm-Breschkin JL, Selleck PW, Usman TB, Johnson MA. Reduced sensitivity of influenza A (H5N1) to oseltamivir. Emerg Infect Dis. 2007;13:1354–7 . 18252107 10.3201/eid1309.07-0164 PMC2857286

[24] Stoner TD, Krauss S, DuBois M, Negovetich J, Stallknecht DE, Senne DA, Antiviral susceptibility of avian and swine influenza virus of the N1 neuraminidase subtype. J Virol. 2010;84:9800–9. 10.1128/JVI.00296-10 20660186 PMC2937791

[25] Kiso M, Ozawa M, Le MT, Imai H, Takahashi K, Kakugawa S, Effect of an asparagine-to-serine mutation at position 294 in neuraminidase on the pathogenicity of highly pathogenic H5N1 influenza A virus. J Virol. 2011;85:4667–72. 10.1128/JVI.00047-11 21367898 PMC3126218

[26] Collins PJ, Haire LF, Lin YP, Liu J, Russell RJ, Walker PA, Crystal structures of oseltamivir-resistant influenza virus neuraminidase mutants. Nature. 2008;453:1258–61. 10.1038/nature06956 18480754

[27] Ilyushina NA, Seiler JP, Rehg JE, Webster RG, Govorkova EA. Effect of neuraminidase inhibitor-resistant mutations on pathogenicity of clade 2.2 A/Turkey/15/06 (H5N1) influenza virus in ferrets. PLoS Pathog. 2010;6:e1000933. 10.1371/journal.ppat.1000933 20523902 PMC2877746

[28] Earhart KC, Elsayed NM, Saad MD, Gubareva LV, Nayel A, Deyde VM, Oseltamivir resistance mutation N294S in human influenza A(H5N1) virus in Egypt. J Infect Public Health. 2009;2:74–80. 10.1016/j.jiph.2009.04.004 20701864

[29] Amaro RE, Swift RV, Votapka L, Li WW, Walker RC, Bush RM. Mechanism of 150-cavity formation in influenza neuraminidase. Nat Commun. 2011;2:388. 10.1038/ncomms1390 PMC3144582 21750542

[30] Hurt AC, Holien JK, Barr IG. In vitro generation of neuraminidase inhibitor resistance in A(H5N1) influenza viruses. Antimicrob Agents Chemother. 2009;53:4433–40. 10.1128/AAC.00334-09 19651908 PMC2764219

[31] Nguyen HT, Fry AM, Loveless PA, Klimov AI, Gubareva LV. Recovery of a multidrug-resistant strain of pandemic influenza A 2009 (H1N1) virus carrying a dual H275Y/I223R mutation from a child after prolonged treatment with oseltamivir. Clin Infect Dis. 2010;51:983–4. 10.1086/656439 20858074

[32] Le MT, Wertheim HF, Nguyen HD, Taylor W, Hoang PV, Vuong CD, Influenza A H5N1 clade 2.3.4 virus with a different antiviral susceptibility profile replaced clade 1 virus in humans in northern Vietnam. PLoS ONE. 2008;3:e3339. 10.1371/journal.pone.0003339 18836532 PMC2556101

[33] Chen H, Smith GJ, Li KS, Wang J, Fan XH, Rayner JM, Establishment of multiple sublineages of H5N1 influenza virus in Asia: implications for pandemic control. Proc Natl Acad Sci U S A. 2006;103:2845–50. 10.1073/pnas.0511120103 16473931 PMC1413830

[34] Boltz DA, Douangngeun B, Phommachanh P, Sinthasak S, Mondry R, Obert C, Emergence of H5N1 avian influenza viruses with reduced sensitivity to neuraminidase inhibitors and novel reassortants in Lao People’s Democratic Republic. J Gen Virol. 2010;91:949–59. 10.1099/vir.0.017459-0 20016036 PMC2888158

[35] Wathen MW, Barro M, Bright RA. Antivirals in seasonal and pandemic influenza-future perspectives. Influenza Other Respitory Viruses. 2013;7:76–80. 10.1111/irv.12049 PMC5978628 23279900

[36] Chan-Tack KM, Gao A, Himaya AC, Thompson EG, Singer ME, Uyeki TM, Clinical experience with intravenous zanamivir under an emergency investigational new drug program in the United States. J Infect Dis. 2013;207:196–8. 10.1093/infdis/jis637 23089591 PMC6657507

[37] White NJ, Webster RG, Govorkova EA, Uyeki TM. What is the optimal therapy for patients with H5N1 influenza? PLoS Med. 2009;6:e1000091. 10.1371/journal.pmed.1000091 19554084 PMC2694988

