# Supplementary material for: Antiviral Susceptibility of Highly Pathogenic Avian Influenza A(H5N1) Viruses Isolated from Poultry, Vietnam, 2009–2011
Source: Emerg Infect Dis. 2013 Dec;19(12):1963–71. doi: 10.3201/eid1912.130705 (PMC3840871; doi:10.3201/eid1912.130705)
Supplement: Technical Appendix 2 — Analysis of highly pathogenic avian influenza A(H5N1) viruses. [file 13-0705-Techapp-s2.pdf]

Article DOI: http://dx.doi.org/10.3201/eid1912.130705

Antiviral Susceptibility of Highly Pathogenic Avian Influenza A(H5N1) Viruses Isolated from Poultry, Vietnam, 2009–2011

Technical Appendix 2

Technical Appendix 2 Table. Epidemiologic information of highly pathogenic influenza avian A(H5N1) viruses collected in Vietnam during 2009–2011 and their GISIAD accession numbers.

	IC50 nmol/L									
					Baseline		Mild outliers§		Extreme outliers¶	
NA inhibitor	Min–Max	Median	Mean ±SD†	Statistical‡ cutoff	No.	Min–Max (fold)#	No.	Min–Max (fold)#	No.	Min–Max (fold)#
Oseltamivir	0.04–527.26	0.44	0.42 ±0.26	2.31	106	0.04–1.39 (1–3)	14	1.49–2.79 (3–6)	5	6.76–527.50 (16–1,227)
Zanamivir	0.13–18.89	0.36	0.36 ±0.14	1.12	118	0.13–1.10 (1–3)	1	1.33 (4)	1	18.89 (54)
Peramivir	0.07–91.22	0.2	0.22 ±0.11	0.74	119	0.07–0.53 (1–3)	0	N/A	1	91.22 (456)
Laninamivir	0.09–2.62	0.23	0.25 ±0.11	0.62	37	0.09–0.53 (1–2)	1	N/A	1	2.62 (10)

*No., number of viruses (including outliers) analyzed to determine the statistical cutoff.
†Mean and SD of IC_{50} values after exclusion of outliers.
‡Determined as U = Q3 + 3*IQR. Interquartile range (IQR) = Q3-Q1. Q1 = 25th percentile; Q3 = 75th percentile.
§Mild outliers, viruses with IC_{50} >U and fold increase <10 times of the median IC_{50};
¶Extreme outliers, viruses with IC_{50} >U and fold differences >10 times the median IC_{50}.
#Fold increase compared to the median IC_{50}.

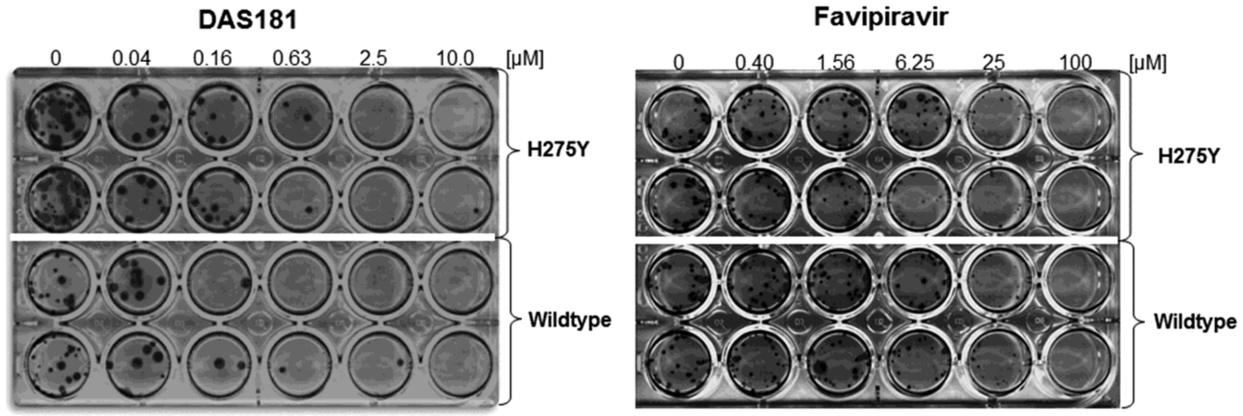

Technical Appendix 2 Figure 1. Inhibition of highly pathogenic avian influenza A(H5N1) virus replication in MDCK-SIAT1 cells in the presence of DAS181 and favipiravir (T-705).

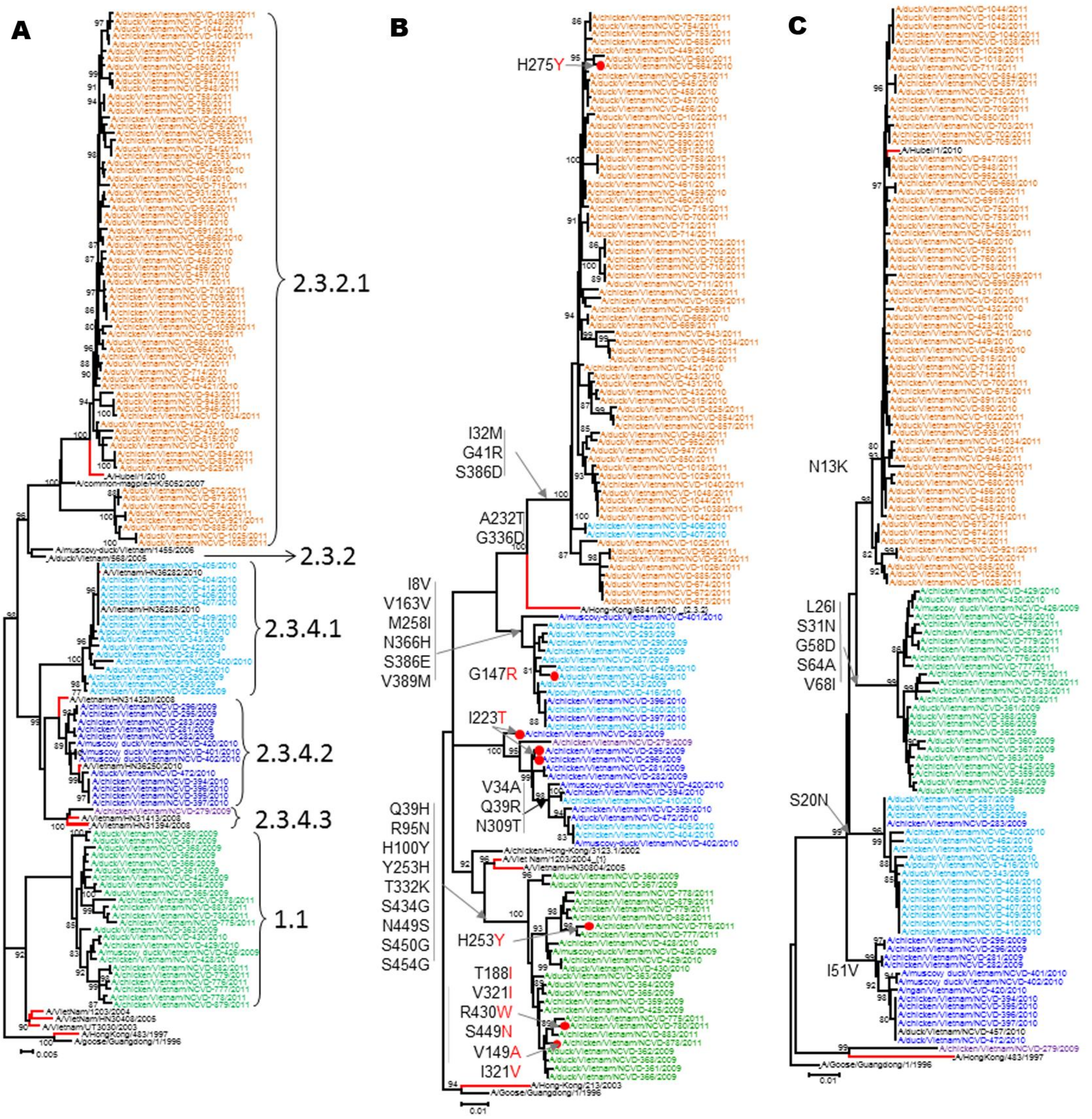

Technical Appendix 2 Figure 2. Phylogenetic analysis of HA (A), NA (B) and M (C) genes of highly pathogenic avian influenza A(H5N1) viruses (n = 125) isolated from poultry in Vietnam during 2009–2011. Phylogenetic trees were generated by the MEGA software package (v5.0) by using the neighbor-joining method with a maximum composite likelihood model. The reliability of phylogenetic inference at each branch node was estimated by the bootstrap method with 1,000 replications by using the MEGA package. Bootstrap values >80% are shown. A/goose/Guangdong/1/1996 (clade 0) was used as a reference for ancestry (root) and numbering. Virus entries are colored in correlation to HA lineage (1.1, light green; 2.3.2.1, orange; 2.3.4.1, blue; 2.3.4.2, dark blue; and 2.3.4.3, purple). Representative H5N1 viruses used

in sequence alignments for tree building are shown in black. Tree branches for human viruses are shown in red; red dots indicate outliers. Scale bars represent nucleotide substitutions per site.

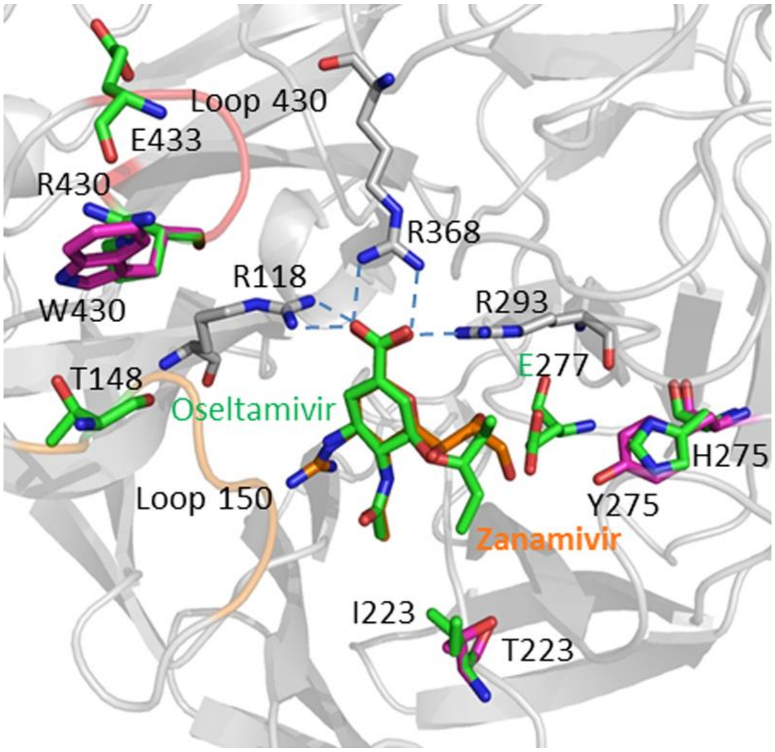

Technical Appendix 2 Figure 3. Visualization of neuraminidase (NA) structure in complex either with oseltamivir (green) or zanamivir (orange) by Pymol software: Effect of NA substitutions including I223T, H275Y, R430W on oseltamivir and/or zanamivir IC_{50} values. Active site residues are displayed in stick form and the backbone is in cartoon form. The loop 150 and 430 were presented for residues 146–152 and 429–432, respectively.
